# Post-Exercise Voluntary Drinking Cessation Is Associated with the Normalization of Plasma Osmolality and Thirst Perception, but Not of Urine Indicators or Net Fluid Balance

**DOI:** 10.3390/nu14194188

**Published:** 2022-10-08

**Authors:** Catalina Capitán-Jiménez, Luis Fernando Aragón-Vargas

**Affiliations:** 1 Department of Nutrition, Universidad Hispanoamericana, San Jose 10101, Costa Rica; 2 Human Movement Science Research Center, Universidad de Costa Rica, San Pedro 11501, Costa Rica

**Keywords:** thirst perception, dehydration, rehydration, voluntary fluid intake, urine osmolality

## Abstract

Post-exercise rehydration has been widely studied, with particular emphasis on retention of ingested fluid; comparatively little research has been conducted on why we drink more or less. To identify physiological values corresponding to voluntary drinking cessation (VDC), nine males exercised intermittently at 70–80% HRmax in the heat (WBGT = 28.1 ± 0.7 °C) to achieve a dehydration of approximately 4.0% body mass (BM). After exercise, participants were instructed to drink water as long and as much as they needed. Urine color (U_color_), specific gravity (USG), osmolality (U_osm_), plasma osmolality (P_osm_), fullness, BM, and thirst perception (TP) were measured pre- and post-exercise and at VDC. Each variable was compared for the three points in time with a one-way ANOVA. Participants reached dehydration of −3.6 ± 0.3% BM. Pre-exercise USG (1.022 ± 0.004) was lower than at VDC (1.029 ± 0.004, *p* = 0.022), U_osm_ did not change over time (*p* = 0.217), and U_color_ was lower pre-exercise (3.4 ± 0.7) vs. post-exercise (5.5 ± 1.23, *p* = 0.0008) and vs. VDC (6.3 ± 1.1, *p* < 0.0001). P_osm_ showed a difference between pre-exercise (289.5 ± 2.3) and post-exercise (297.8 ± 3.9, *p* = 0.0006) and between post-exercise and VDC (287.3 ± 5.4, *p* < 0.0001). TP post-exercise (96.4 ± 4.34) was significantly higher than pre-exercise (36.2 ± 19.1) and VDC (25.0 ± 18.2, *p* < 0.0001). At VDC, participants had recovered 58.7 ± 12.1% of BM loss. At the point of voluntary drinking cessation, P_osm_ and thirst perception had returned to their pre-exercise values, while rehydration relative to initial BM was still incomplete.

## 1. Introduction

It has been shown that the first 3–6 h post-exercise induced dehydration, as subjects tend to stop drinking before restoring all their body fluid deficit. However, little research has been conducted on why we stop drinking during rehydration. Combined changes in body weight and volume consumption are the most common measure of exercise-related dehydration [1,2,3], but there are also other ways to measure dehydration. Some of them involve laboratory techniques such as urine and plasma osmolality [2,4], while others are more practical, such urine specific gravity (USG) and urine color [5,6]. Some of these methods have been used to study thirst and hypohydration at rest; however, there is insufficient information about how these measures behave following post-exercise rehydration, in particular, at the point when drinking stops.

Why humans stop drinking at some point after exercise is not clear yet. Drinking cessation is largely attributed to each individual’s thirst or to thirst perception once the exercise is over [7]. There is evidence to support the claim that people do not recover the weight they lose through sweating if they drink to thirst perception during exercise; the same thing happens during post-exercise rehydration [8,9,10,11]. On the other hand, some evidence suggests that it is not necessary to match fluid losses resulting from sweating, and that ingesting liquids according to thirst perception allows for the recovery of physiological variables such as plasma osmolality [7,12].

Thirst has been shown to increase consistently as dehydration progresses during exercise in the heat [13], while it quickly attenuates after water ingestion [9,14]. Plasma osmolality shows the same behavior: it increases with acute dehydration and diminishes with rehydration [4]. Some researchers claim that plasma osmolality (P_osm_) and thirst dictate when someone has drunk enough liquids after exercise [7,12], but they do not present reference values of thirst perception or plasma osmolality at which humans decide to stop drinking. 

As for urinary markers, even though a late response to fluid intake has been reported [4,15], these could be helpful, practical indicators of the person’s hydration status. There is evidence to support urinary markers as good indicators of hypohydration: when humans are hypohydrated, urine color increases (becomes darker), and urine osmolality and specific gravity also increase. In the presence of hyperhydration, the values for all of these urinary markers decrease [16,17]. What is not known is how they behave dynamically, particularly at the point of drinking cessation, when humans drink *ad libitum* after exercise-induced dehydration. Drinking cessation may occur when one or more of the preceding physiological variables return to normal (euhydration) values.

Knowing that thirst is a mechanism that physically active people are familiar with, it is essential to be clear about its role in recovering from dehydration after exercising. As mentioned above, there is literature that powerfully demonstrates that the perception of thirst disappears once people drink fluids, even if dehydration is still present [1,14,17]. The response of thirst perception is complicated by the fact that it is affected by multiple factors [18,19], especially when studying the perception of post-exercise thirst; one of the factors to take into account is whether the amount of liquid that can be ingested before feeling full is enough to recover what is lost during exercise [20]. The type of liquid can also affect fluid replacement after exercise [4], since electrolyte and carbohydrate content in beverages can affect the perception of thirst, and even the osmolality response of plasma [21]. However, no studies present evidence or data showing what physiological values have returned to their initial values at the moment that people decide they do not want to ingest more liquid. This is a valuable question because it can help in the understanding of post-exercise rehydration: whether thirst is an effective or imperfect mechanism to gauge how much fluid is needed after exercise-induced dehydration. 

Therefore, this study aimed to identify the values of plasma osmolality, urinary markers, thirst perception, stomach fullness, and net fluid balance at the point of voluntary drinking cessation, and to compare them with the corresponding pre-exercise values for humans drinking water ad libitum after exercise-induced dehydration. This is an exploratory descriptive study to better understand what signals may be inducing humans to stop drinking. 

## 2. Materials and Methods

Nine healthy, physically active males provided written informed consent prior to participation in this study, as approved by the Institution’s Ethics and Science Committee, document number VI 57292013.

After an overnight fast, each participant arrived in the laboratory, performed the baseline procedures, exercised in the heat, and rehydrated ad libitum in a single visit. At different points during the protocol, physiological measurements were made; self-reports were also obtained for thirst, stomach fullness, heat perception, and Exercise-Related Transient Abdominal Pain, or ETAP [22]. ETAP is also known as “stich” or “side-ache”, the sharp abdominal pain occasionally associated with intense exercise. 

Participants reported to the laboratory on testing day and voided their bladders completely. Urine was collected and analyzed with a refractometer for urine specific gravity (ATAGO^®^ model URC-Ne, Minato-ku, Tokyo, Japan). Blood samples were obtained from an antecubital vein after the participants rested in a sitting position for a minimum of 5 min. Urine (U_osm_) and plasma osmolality (Posm) were measured via freezing point depression (Advanced Instruments 3250 osmometer; Norwood, MA, USA). Baseline body weight was measured with each participant nude and dry (e-Accura^®^ scale, model DSB291, Qingpu, Shanghai, China) to the closest 10 g. 

Baseline self-reported thirst was recorded with a visual analog scale, which consisted of marking a continuous line of 100 mm, on the left end indicating “not thirsty at all” and on the right “extremely thirsty” [23,24]. A 0 to 8 scale was used for heat perception [25], in which 0 corresponds to “incredibly cold” and 8 to “incredibly hot”. Finally, the feeling of stomach fullness and ETAP the questions were: “how full do you feel?” and “How much ETAP do you feel?”, respectively, with a score between 1 (not at all, none) and 5 (very, too much). These scales were adapted from [22]. To avoid cross-contamination between answers, each participant was asked to count down from 40 to 0 in multiples of 5; randomization of the order of presentation of the questions was also used for each moment and each person.

After baseline measurements, each participant ingested a standardized breakfast (750 kilocalories: 24.6% fat, 20.7% protein, and 54.7% carbohydrate; 250 mL of fluid, 1500 mg sodium). The pre-exercise measurements were made after resting for thirty minutes and then the exercise session started. The subjects exercised intermittently (30 min cycle ergometry—30 min treadmill running) at 70–80% of maximum predicted heart rate in the heat (WBGT = 28.1 ± 0.7 °C; T = 34.9 ± 0.8 °C; RH = 72 ± 3%). Nude and dry body weight was taken every 30 min to monitor their fluid losses until reaching approximately 4% body mass (BM) loss. Water ingestion during exercise was not allowed. Environmental stress was monitored with a Questemp36^®^ monitor (3M, Oconomowoc, WI, USA).

Upon exercise termination and after measuring all post-exercise values, participants were instructed to drink as much and for as long as they needed from previously weighed bottles. They did not know that the experiment would end when they stopped drinking water; in addition, during recruitment, participants were told that the protocol would last two hours longer than estimated by the researchers. This helped assure that participants would not truncate voluntary drinking for other reasons not related to their perceived hydration needs. The weight of water bottles was monitored with a food scale, every 15 min. Urine color (U_color_), specific gravity (USG) and osmolality (U_osm_), plasma osmolality (P_osm_), fullness, body mass, and thirst perception (TP) were measured pre- and post-exercise and post-rehydration, at the point of voluntary drinking cessation (VDC); heat perception (HP) and ETAP were measured at the same points and used as distractors. Pre-exercise minus post-exercise body mass or VCD was used to calculate net fluid balance (NFB). The point of drinking cessation was set as the moment when water intake was less than 100 mL in a 15 min period. This point was set based on the timeline of rehydration reported in [14,26].

Mean and standard deviation were used for descriptive statistics. One-way analyses of variance were performed to test for differences over time for each variable (urine and plasma osmolality, urine color, thirst, fullness, net fluid balance, and USG). When ANOVA showed a statistically significant main effect, Tukey’s post hoc tests were performed to compare group differences.

## 3. Results

Nine males completed the study: age = 26.6 ± 3.4 years, height = 1.78 ± 0.07 m, body mass = 82.65 ± 12.64 kg, baseline USG = 1.021 ± 0.004, fullness = 1.2 ± 0.4, Uosm = 852 ± 156, urine color = 3.3 ± 0.7, and thirst = 43 ± 26 mm. The volunteers reached a dehydration equivalent to 3.6 ± 0.3% BM in 121.7 ± 19.0 min of intermittent exercise. 

**Figure 1:**
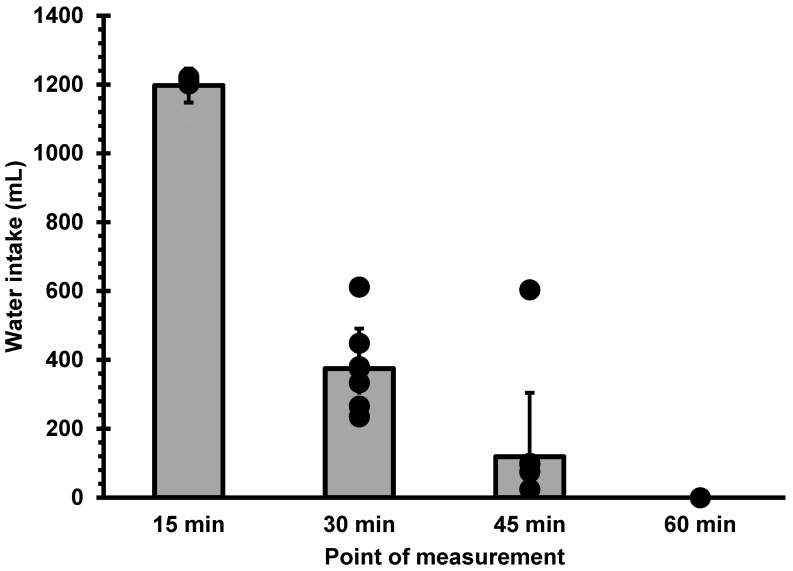
Voluntary water intake for each 15 min period. Bars represent average consumption and dots represent individual values of consumption. Only one participant did not interrupt rehydration at the 45 min point, but his intake from 45 to 60 min was negligible.

Average voluntary water intake post-exercise was 1691 ± 290 mL (20.93 ± 3.91 mL/kg) in 46.7 ± 5 min. The highest intake was 2427 mL (29.01 mL/kg) and the lowest was 1445 mL (16.76 mL/kg). Figure 1 shows average water intake every 15 min. Figure 1 also shows the individual values of water consumption at each measurement point. These individual values are expressed as percentage of total intake in Table 1. Total fluid consumption led to partial rehydration of 58.7 ± 12.1% (min 55%, max 86%).

**Figure 2:**
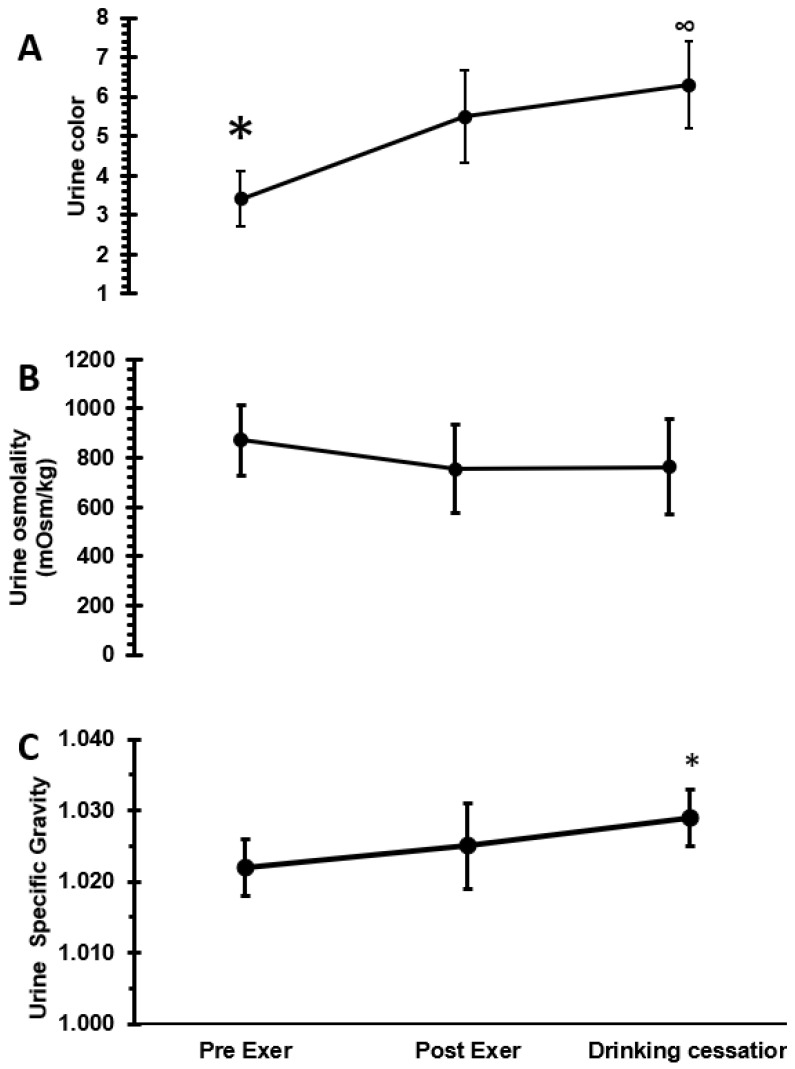
(**A**) ANOVA (F = 18.25, *p* < 0.0001) for urine color, * difference between pre- and post-exercise (dehydration) (*p* = 0.0008), post-exer (dehydration) and drinking cessation (*p* = 0.259), ∞ pre-exer and drinking cessation (*p* < 0.0001). (**B**) ANOVA for urine osmolality. There was no effect of time (F = 1.62, *p* = 0.217). (**C**) ANOVA (F = 18.25, *p* < 0.0001) for urine color, * difference between pre- and post-exercise (dehydration) (*p* = 0.0008), post-exer (dehydration) and drinking cessation (*p* = 0.259), pre-exer and drinking cessation (*p* < 0.0001). (**C**) ANOVA (F = 4.14, *p* = 0.028) for urine specific gravity. * Statistical difference between pre-exer and drinking cessation (*p* = 0.022).

Regarding USG, there was a significant change over time (F = 4.14, *p* = 0.028): USG at drinking cessation was higher (1.029 ± 0.004) than pre-exercise (1.022 ± 0.004, *p* = 0.022) but not different between pre- and post-exercise (1.025 ± 0.006) nor post-exercise and drinking cessation (*p* = 0.447 and *p* = 0.248, respectively) (see Figure 2).

Figure 2 shows the analysis for urine osmolality, indicating no significant difference over time (pre: 870.7 ± 142.9, post: 754.8 ± 177.7, and cessation: 763.7 ± 193.9; F = 1.62, *p* = 0.217) and urine color change over time (F = 18.25, *p* < 0.0001). Pre-exercise was lower (3.4 ± 0.7) than post-exercise (5.5 ± 1.2; *p* = 0.0008), post-exercise and drinking cessation (6.3 ± 1.1) were not different (*p* = 0.276), and drinking cessation was higher than pre-exercise (*p* < 0.0001).

**Figure 3:**
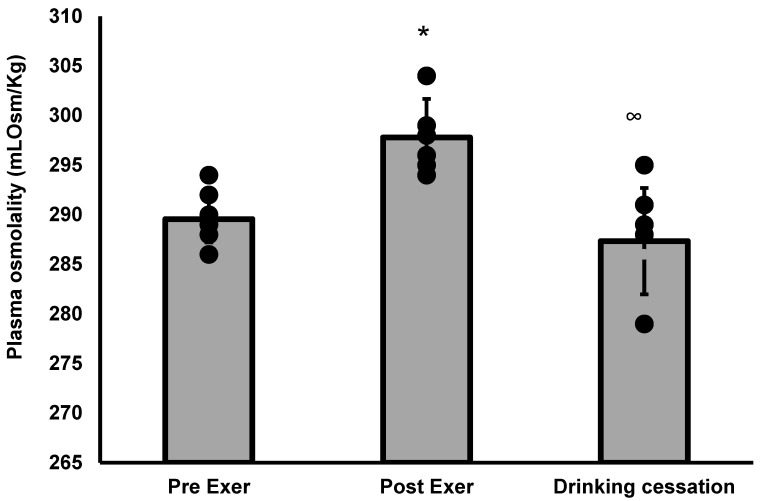
ANOVA analysis for plasma osmolality, * pre-exer vs. post-exer (dehydration) (*p* = 0.0006), pre-exer vs. drinking cessation (*p* = 0.524), ∞ post-exer (dehydration) vs. drinking cessation (*p* < 0.0001). Bar height represents average values; dots represent individual plasma osmolality.

The ANOVA (F = 16.66, *p* = 0.0001) for plasma osmolality shows a difference between pre-exercise (289.5 ± 2.3) and post-exercise (297.8 ± 3.9) (*p* = 0.0006), but not between the former and drinking cessation (287.3 ± 5.4) (*p* = 0.524). There is also a significant difference between post-exercise and drinking cessation (*p* < 0.0001) See Figure 3.

**Figure 4:**
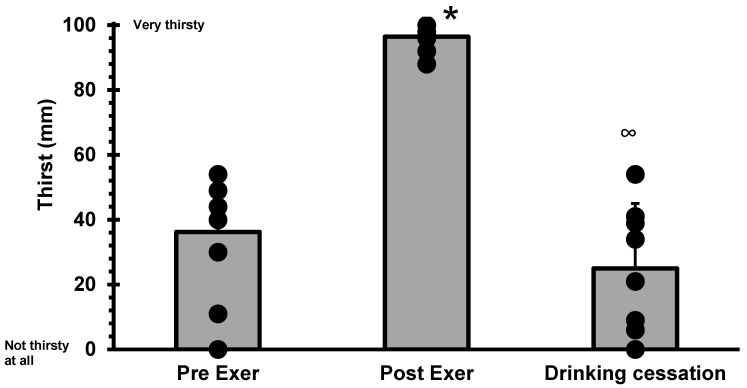
ANOVA analysis of thirst perception, no difference pre- vs. drinking cessation (*p* = 0.289), * pre-exer vs. post-exer (dehydration) (*p* < 0.0001), ∞ post-exer (dehydration) vs. drinking cessation (*p* < 0.0001). Bar height represents average values; dots represent individual thirst perception.

Thirst perception ANOVA shows a significant difference over time (F = 56.59, *p* < 0.0001). Post-exercise thirst (96.4 ± 4.34) was significantly higher than both the pre-exercise (36.2 ± 19.1; *p* < 0.0001) and the drinking cessation (25.0 ± 18.2; *p* < 0.0001). However, thirst values of pre-exercise and at drinking cessation did not differ (*p* = 0.289) (see Figure 4).

**Figure 5:**
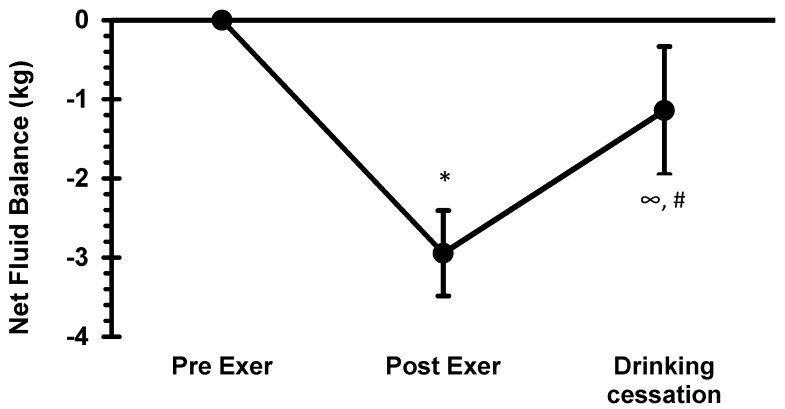
ANOVA analysis for net fluid balance * pre vs. post-exer (*p* < 0.0001) ∞ post-exercise (dehydration) vs. drinking cessation (*p* < 0.0001) # pre-exer vs. drinking cessation (*p* = 0.0012). Mean ± SD.

Net fluid balance (Figure 5) ANOVA (F = 57.97, *p* < 0.0001) shows statistically significant differences among measures of pre- (0 ± 0) and post-exercise (−2.94 ± 0.57; *p* < 0.0001), post-exercise and drinking cessation (1.14 ± 0.86; *p* < 0.0001), and pre-exercise and drinking cessation (*p* = 0.0012).

**Figure 6:**
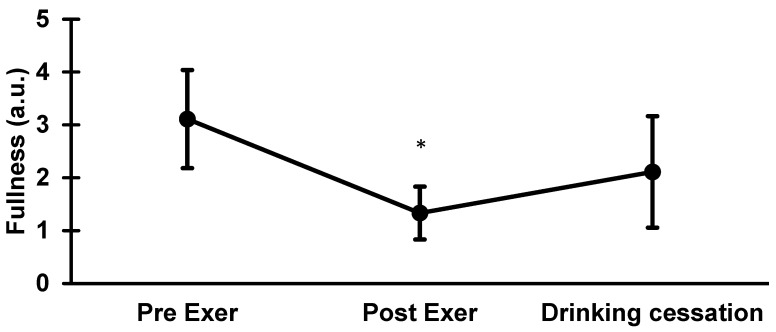
ANOVA analysis for fullness. Pre-exer and drinking cessation point (*p* = 0.055), pre-exer vs. post-exer (dehydration) (*p* = 0.0012), post-exer (dehydration) vs. drinking cessation (*p* = 0.251). * *p* > 0.05.

ANOVA (F = 8.44, *p* = 0.0017) for stomach fullness showed no difference between pre-exercise (3.1 ± 0.9) and drinking cessation point (2.1 ± 1.1; *p* = 0.055). Pre-exercise and post-exercise (1.3 ± 0.5) were different (*p* = 0.0012), but there was no significant difference between post-exercise and drinking cessation (*p* = 0.251) See Figure 6. 

## 4. Discussion

This study identified the values of plasma osmolality, urinary markers, thirst perception, stomach fullness, and net fluid balance at the point of voluntary drinking cessation when humans drank water ad libitum following exercise-induced dehydration and compared them with the corresponding pre-exercise values. When these values matched statistically, that physiological variable could be considered a possible trigger of the drinking cessation; nevertheless, it is essential to note that not every variable changed significantly at the post-exercise point. Our results show that voluntary drinking cessation coincided with values equal to or lower than pre-exercise values of P_osm_ and thirst perception after a significant increase at the post-exercise point. Meanwhile, the net fluid balance was markedly reduced at the post-exercise point but had not returned to the pre-exercise value at the point of drinking cessation. Finally, U_color_, USG, and stomach fullness did not show the expected behavior, because their values had not returned to pre-exercise levels after a significant change vs. post-exercise. The authors expected that participants might stop drinking if they became full, but fullness values were relatively low at the time of drinking cessation. U_osm_ values did not suffer any change during the experiment. There is more discussion on these urine variables below.

In this study, at the point of drinking cessation (mean 46.7 min), plasma osmolality had already returned to pre-exercise values, even though subjects had recovered only 58% of the sweat they lost. Allen et al. [4] found the same P_osm_ behavior, and this may be explained because the human body is designed to defend P_osm_. Lieb et al. [27] found similar results to those in our study, and they hypothesized that this might be because when blood osmolality and volume are placed in conflict, osmolality defense is prioritized. Popowski et al. [15] mentioned that P_osm_ identifies a state of euhydration and is sensitive to changes in hydration status but lags during periods of rapid body fluid turnover. Our study does not support this slow response.

The effect of returning to pre-exercise values was not found on such urinary indices as USG and urine color, in line with some studies showing that USG lags behind P_osm_ [15,28]. In the present study, pre-exercise values for USG were a little higher (1.022 ± 0.004) than the usual cutoff point (1.020); nevertheless, USG behaved almost identical to studies where participants start exercise below 1.020 [15,28]. Those indices present the expected behavior in pre- and post-exercise, but the values for drinking cessation were higher than those for post-exercise; we expected a decrease due to the volume of water ingested. This may be due to the short time of rehydration: our subjects ingested water for an average of 46.7 min and then provided a urine sample, which could be too early to see a recovery in these urinary indices. U_osm_ did not show any changes, suggesting that it is an insensitive, and therefore useless, variable. Hew-Buttler [12] obtained a similar response with 2% dehydration but allowed the participants to ingest liquid during the exercise session. Finally, USG and U_osm_ in the present study do not show a parallel behavior over time, as has been shown in the literature [5,15]. An extreme value in the present U_osm_ data pre-exercise (1046 mOsm∗kg^−1^) was identified for one subject, which would explain the different behavior of this variable. 

Some studies suggest that drinking to thirst protects plasma osmolality, and this may indicate that athletes were drinking adequate amounts of fluid in response to osmotic thirst stimulation [7,29]. Plain water is particularly effective at diminishing plasma osmolality [21]. However, it is known that it does not guarantee good rehydration [30]. Studies have shown that drinking sodium-containing beverages improves post-exercise fluid retention, thus helping to recover fluid imbalance caused by dehydration [30,31]. In this study, we used plain water because it allows for testing the effect of fluid volume on thirst perception without the confounding effects of electrolytes and other solutes. The response obtained in the perception of thirst coincides with that from Peyrot et al. [32], who found that cold water decreases thirst. Our study found that thirst perception increased during exercise when people could not drink, but post-exercise thirst perception was almost at the highest point of the scale (96.4 ± 3.8). After only 46.7 min of voluntary rehydration, thirst perception was slightly lower (not statistically different) than pre-exercise. Our results are not different from other studies [9,10,14]. Furthermore, Lieb et al. [27] mention that drinking can quench thirst within seconds, long before the ingested water has had time to alter the blood volume or osmolality. Apparently, thirst is a valuable signal for the need for fluid intake during exercise while no drinking is permitted [13,17], but once water drinking begins the thirst stimulus is disturbed: in the present study it is interesting to note that even when thirst perception and P_osm_ indicated that subjects had drunk “enough”, at the point of drinking cessation, recovery was only 58% of the lost weight from fluid loss.

The authors acknowledge that although average thirst perception values at drinking cessation (25 mm) were not statistically different from the average pre-exercise thirst value (36 mm); the variability in the responses at the moment of VCD was very large (SD = 18.2). Nevertheless, it must be pointed out that pre-exercise variability was also very large (SD = 19.2)

Other factors affect thirst perception, such as environmental heat and humidity or the stimulation of oropharyngeal receptors. The latter has been studied by mouth rinsing protocols with different solutions. Best et al. [33] used such a protocol with water, carbohydrate 10% maltodextrin, combined carbohydrate and menthol, and menthol, finding no significant differences in the perception of thirst except for a menthol beverage, which moderately reduced thirst. The design of the present study is not amenable to this type of analysis; however, future studies could incorporate mouth rinsing protocols in combination with voluntary water intake to better understand thirst and hydration. The same protocol of the present study could also be repeated with different beverages.

The authors hypothesized that participants might stop drinking because they became full, but a different response was found: average fullness reported by participants was 2.1 at the moment of drinking cessation, which is rather low. This result coincides with Engell et al. [20], who found that the feeling of fullness was only relevant at the highest level of dehydration (7% BM), while showing only moderate changes with the intermediate level of dehydration. In the present study, the participants ingested a large amount of liquid (mean = 1200 mL) in a short period (15 min), which must have caused a feeling of high fullness between 0 and 15 min (not measured). High stomach volume causes a faster gastric emptying [31,34]. Voluntary fluid intake, however, remained high at the 30 and 45 min time points. These results warrant further research to determine whether stomach fullness is associated with voluntary water intake during post-exercise rehydration.

The authors wish to acknowledge that there are two things in this study design that could be improved in a follow-up study, now that the major changes have been confirmed, to better understand the dynamics of physiological values: the first is to try to record plasma osmolality more frequently, every 5, 10, or 15 min; the second is to collect thirst perception and fullness data every 15 min during rehydration. This will allow a better understanding of the regulatory responses of plasma osmolality and its relationship with fluid intake.

## 5. Conclusions

In conclusion, this study suggests that the moment of drinking cessation is associated with the return to pre-exercise values of plasma osmolality (287.3 ± 5.4) and thirst perception (25.0 ± 18.2). The return of these variables happened in approximately 46.7 min (maybe less) of voluntary water intake after subjects had recovered an average of 58% of their body weight loss.

This research presents a novel design showing the values of blood and urinary variables at the moment when subjects voluntarily decide to stop fluid intake after dehydration caused by exercise. In addition, it presents data on the behavior of plasma osmolality and how it can return to normal values even when fluid intake is considerably lower than fluid loss due to exercise.

## Figures and Tables

**Table 1:** Individual percentage of total intake and mL/kg every 15 min.

Subject	Time	Body Weight (kg)	Intake (mL)	mL/kg	%
1	15	70.91	1217	17.2	72.31
	30	71.95	441	6.1	26.20
	45	72.50	25	0.3	1.49
2	15	81.50	1211	14.9	49.90
	30	83.12	612	7.4	25.22
	45	83.67	604	7.2	24.89
3	15	84.50	1066	12.6	73.77
	30	86.00	282	3.3	19.52
	45	86.20	97	1.1	6.71
4	15	108.62	1214	11.2	69.85
	30	110.14	449	4.1	25.83
	45	110.18	75	0.7	4.32
5	15	79.95	1222	15.3	73.88
	30	81.39	334	4.1	20.19
	45	81.48	98	1.2	5.93
6	15	65.53	1215	18.5	78.39
	30	66.92	235	3.5	15.16
	45	67.60	100	1.5	6.45
7	15	75.40	1213	16.1	80.71
	30	77.99	266	3.4	17.70
	45	78.37	24	0.3	1.60
8	15	77.88	1216	15.6	75.02
	30	79.25	381	4.8	23.50
	45	79.45	24	0.3	1.48
9	15	70.91	1217	17.2	75.03
	30	76.84	376	4.9	23.47
	45	77.14	24	0.3	1.50

## Data Availability

Raw data for this research are available in the institutional repository Kerwa: https://hdl.handle.net/10669/85305 (accessed on 22 August 2022).
